# A Rare Case of Necrotizing Sarcoid Granulomatosis Involving Liver

**DOI:** 10.7759/cureus.5366

**Published:** 2019-08-11

**Authors:** Haisam Abid, Nadir Siddiqui

**Affiliations:** 1 Internal Medicine, Bassett Medical Center, Cooperstown, USA

**Keywords:** granulomas, sarcoid, liver

## Abstract

Sarcoidosis is a multi-system inflammatory disease, characterized by formation of non-caseating epithelioid granulomas. It usually involves lungs, lymph nodes, skin, joints, eyes and uncommonly liver. Necrotizing sarcoid granuloma is a rare entity. We are presenting a case of necrotizing sarcoid granuloma of liver which is extremely rare.

## Introduction

Sarcoidosis is a non-caseating granuloma disease involving multiple organ systems. It is more frequently presented as hilar lymphadenopathy and pulmonary parenchymal disease. Skin, joints and eyes are other organs that are generally influenced by sarcoidosis. Non-caseating liver sarcoidosis is an unusual disease which can be asymptomatic or can lead to cholestasis, portal hypertension, cirrhosis or liver failure. One case series showed that steroids were not much beneficial in the treatment of liver sarcoidosis [1]. Necrotizing sarcoid granulomatosis is a rare condition and has been reported to involve most commonly lungs [2]. We are presenting a case of necrotizing sarcoid granulomatosis involving the liver, finding of which is extremely rare. Our case also demonstrates that prednisone can be used to treat this disease.

## Case presentation

**Figure 1:**
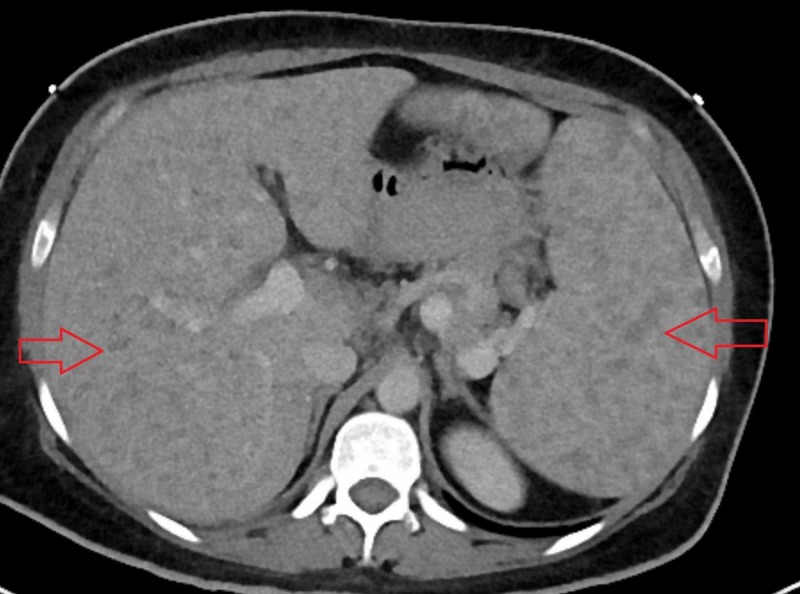
Computed tomography (CT) scan of the abdomen and pelvis with intravenous contrast showing multiple nodules in liver and spleen.

A 37-year-old female came to the hospital with complaint of right upper quadrant abdominal pain associated with high-grade fever, shaking chills, nausea and vomiting for four days. She also reported 70 pounds weight loss over four months duration. On physical examination, she was febrile with temperature of 101 F, tachycardic but normotensive. Her abdomen was soft, tenderness present in right upper quadrant without guarding, rigidity or organomegaly. Lab workup showed that she was leukopenic, 3.7 x 109 cells/L (normal range: 3.7-11 x 109 cells/L). She also had mild elevation of alkaline phosphatase. Other liver enzymes were within normal limit. Human immunodeficiency virus (HIV) serology was negative. After obtaining blood culture, the patient was treated with broad-spectrum antibiotic for sepsis related to intra-abdominal pathology. Her symptoms did not improve. Subsequently, the patient had computed tomography (CT) scan of the chest, abdomen and pelvis with contrast which revealed innumerable lesions scattered throughout in liver and spleen (Figure 1).

**Figure 2:**
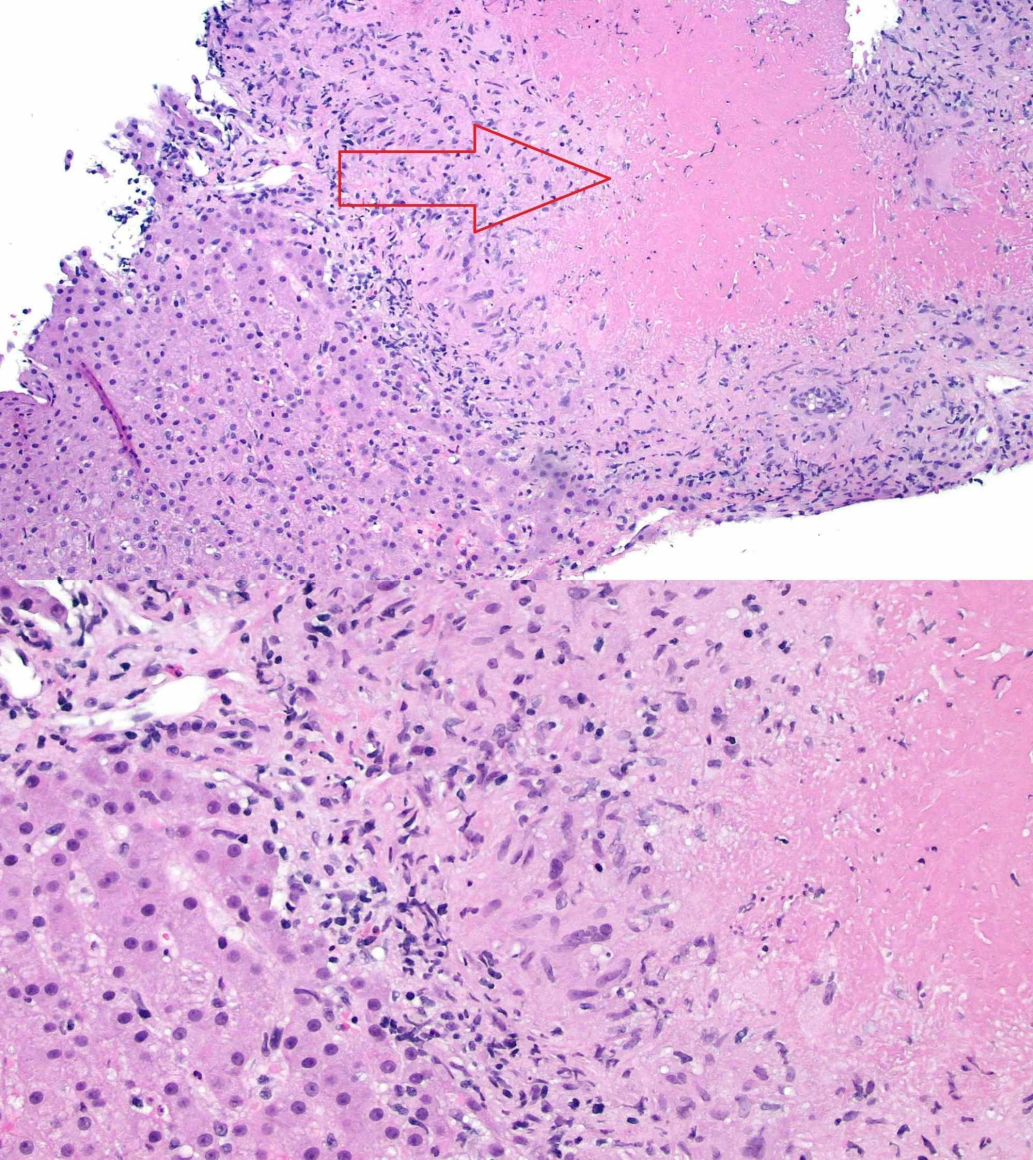
Necrotizing granulomata within the liver.

Ultrasound-guided liver biopsy was done which showed necrotizing granulomas and negative for fungal, mycobacterial or other bacterial cultures. Serology was also negative for fungitell, histoplasma, antineutrophil cytoplasmic antibodies (ANCA) and QuantiFERON gold was also negative (Figure 2).

The patient was diagnosed with necrotizing granulomas secondary to liver sarcoidosis and was started on prednisone after which her symptoms subsided. She is doing well on follow-up outpatient visits.

## Discussion

Sarcoidosis is an inflammatory disorder, the exact etiology is unknown. It is characterized by the presence of non-caseating granulomas [3]. It most commonly affects lungs but sometimes it can affect multiple organs of the body, including the liver. The majority of patients are asymptomatic but up-to 50% to 60% of patients having granulomas found on liver biopsy [4]. The peak age of occurrence is 20-40 years, as with inflammatory disorders it affects female more than males. Sometimes, second age peak >50 years has also been observed [5].

Most common symptoms associated with hepatic sarcoidosis include right upper quadrant pain, pruritis, fatigue and jaundice. Rarely, it can cause life-threatening complications such as portal hypertension and cirrhosis. More than half of the patients are asymptomatic despite radiographic and even with biopsy proven disease [2]. The distinctive characteristic feature of sarcoidosis is the formation of epithelioid granulomas that are typically non-caseating, in the absence of tuberculosis, fungal infection, malignancy, or other causes of granulomatous reactions [6].

Elevations of alkaline phosphatase (ALP) and gamma glutamyltranspeptidase are indications of liver and biliary involvement. ALP can be elevated five to ten times of upper limit of normal as seen in our patient. Alanine aminotransferase (ALT) and aspartate aminotransferase (AST) elevations are usually mild and less significant as compared to ALP. The severity of hepatic sarcoidosis is directly linked with abnormalities in liver function tests [4]. Computed tomography (CT) scan of abdomen or ultrasonography should be obtained if there is suspicion of hepatic sarcoidosis; most common radiographic findings include hepatomegaly and diffuse hypoattenuated nodules in liver. These nodules can be confused with malignancy or other granulomatosis diseases. The concomitant presence of nodules in spleen is more suggestive of sarcoidosis as seen in our patient [7].

Liver biopsy should be considered if physician is unsure about the diagnosis, as was done with our patient. Histopathological examination is the gold standard diagnostic test. In sarcoidosis, multiple noncaseating and epithelioid granulomas are seen in the periportal and the portal regions of the liver. Sarcoid epithelioid granulomas are characterized by macrophages that form giant cells surrounded by fibrin rings [2-4]. Long standing disease can cause cirrhotic changes in the liver [8].

There is limited data on the literature review regarding the treatment of hepatic sarcoidosis given the rarity of this condition. In asymptomatic patients or mild elevation of liver enzymes with normal synthetic liver function observation alone is sufficient. But these patients would require close monitoring as our patient. It has been noted that in some asymptomatic patients, abnormal serum liver tests can resolve spontaneously or remain stable for many years. Steroids and ursodeoxycholic acid should be considered in symptomatic patients, and have biochemical evidence of cholestasis or who are at high risk of developing hepatic complications such as portal hypertension or cirrhosis [2].

Corticosteroids can decrease the number of hepatic granulomas by suppression of the inflammatory response and reduce hepatomegaly, which can resolve or decrease abdominal pain. In addition, steroids are recommended for patients with constitutional symptoms such as fever, fatigue, pruritus, and weight loss. Low-dose prednisone may be enough for those with mild symptoms, while a higher dose of prednisone should be considered for those with severe symptoms [2-9].

Treatment duration should be determined by clinical and laboratory response. Some authors recommend 12 months of therapy before decreasing or tapering steroid dosage. Relapsing symptoms may require long-term therapy or steroid-sparing agents. Immunosuppressive agents such as azathioprine, cyclophosphamide, methotrexate or infliximab may be useful for patients for whom prednisone fails or patients who are deemed to be steroid dependent [2].

## Conclusions

Hepatic sarcoidosis is a rare granulomatous disease of unknown etiology. Seventy percent of patients have epithelioid non-caseating granulomas on liver biopsy; around 30% of patients have hepatomegaly or transaminitis. Hepatic sarcoidosis is mainly asymptomatic and requires no treatment. Pain in the right upper quadrant of the abdomen, fatigue, pruritus, and jaundice are the most commonly associated symptoms. For symptomatic patients, prednisone and ursodeoxycholic acid may be considered. Those patients who do not respond to steroids and ursodeoxycholic acid may require other immunosuppressive agents. Portal hypertension and cirrhosis are possible complications from long-standing hepatic sarcoidosis.
